# *Ralstonia solanacearum* Type III Effector RipAY Is a Glutathione-Degrading Enzyme That Is Activated by Plant Cytosolic Thioredoxins and Suppresses Plant Immunity

**DOI:** 10.1128/mBio.00359-16

**Published:** 2016-04-12

**Authors:** Takafumi Mukaihara, Tadashi Hatanaka, Masahito Nakano, Kenji Oda

**Affiliations:** Research Institute for Biological Sciences, Okayama (RIBS), Yoshikawa, Okayama, Japan

## Abstract

The plant pathogen *Ralstonia solanacearum* uses a large repertoire of type III effector proteins to succeed in infection. To clarify the function of effector proteins in host eukaryote cells, we expressed effectors in yeast cells and identified seven effector proteins that interfere with yeast growth. One of the effector proteins, RipAY, was found to share homology with the ChaC family proteins that function as γ-glutamyl cyclotransferases, which degrade glutathione (GSH), a tripeptide that plays important roles in the plant immune system. RipAY significantly inhibited yeast growth and simultaneously induced rapid GSH depletion when expressed in yeast cells. The *in vitro* GSH degradation activity of RipAY is specifically activated by eukaryotic factors in the yeast and plant extracts. Biochemical purification of the yeast protein identified that RipAY is activated by thioredoxin TRX2. On the other hand, RipAY was not activated by bacterial thioredoxins. Interestingly, RipAY was activated by plant *h*-type thioredoxins that exist in large amounts in the plant cytosol, but not by chloroplastic *m*-, *f*-, *x*-, *y*- and *z*-type thioredoxins, in a thiol-independent manner. The transient expression of RipAY decreased the GSH level in plant cells and affected the flg22-triggered production of reactive oxygen species (ROS) and expression of pathogen-associated molecular pattern (PAMP)-triggered immunity (PTI) marker genes in *Nicotiana benthamiana* leaves. These results indicate that RipAY is activated by host cytosolic thioredoxins and degrades GSH specifically in plant cells to suppress plant immunity.

## IMPORTANCE

*Ralstonia solanacearum* is the causal agent of bacterial wilt disease of plants. This pathogen injects virulence effector proteins into host cells to suppress disease resistance responses of plants. In this article, we report a biochemical activity of *R. solanacearum* effector protein RipAY. RipAY can degrade GSH, a tripeptide that plays important roles in the plant immune system, with its γ-glutamyl cyclotransferase activity. The high GSH degradation activity of RipAY is considered to be a good weapon for this bacterium to suppress plant immunity. However, GSH also plays important roles in bacterial tolerance to various stresses and growth. Interestingly, RipAY has an excellent safety mechanism to prevent unwanted firing of its enzyme activity in bacterial cells because RipAY is specifically activated by host eukaryotic thioredoxins. This study also reveals a novel host plant protein acting as a molecular switch for effector activation.

## INTRODUCTION

*Ralstonia solanacearum* is a Gram-negative soilborne bacterium that causes bacterial wilt in more than 200 plant species (1). This pathogen enters the plant roots through natural openings and wound sites and proliferates in the vascular system. The bacterium secretes large amounts of extracellular polysaccharides (EPS) and cell-wall-degrading enzymes inside plants, which block water transport and destroy the vascular system, causing severe wilting and death of infected plants (2, 3).

Plants have evolved two major defense systems to protect themselves from infection by diverse pathogens (4). One is the pathogen-associated molecular pattern (PAMP)-triggered immunity (PTI), through which potential pathogens are recognized by detecting their components through the pattern recognition receptors (PRRs) on the surface of plant cells (5). Induction of PTI leads to a wide variety of disease resistance responses, including activation of mitogen-activated protein kinases (MAPKs) and calcium-dependent protein kinases (CDPKs), generation of reactive oxygen species (ROS), expression of defense-related genes, production of defense hormones, synthesis of phytoalexin, and deposition of callose (6, 7). To overcome host PTI and to succeed in infection, pathogens have evolved to produce effector proteins that interfere with PTI. To counter effector-mediated suppression of PTI, plants have evolved to have another system, called effector-triggered immunity (ETI), that recognizes true pathogens by directly or indirectly detecting pathogen-secreted effectors through the disease-resistance (R) proteins inside plant cells (8). ETI induces very strong and more prolonged immune responses compared with PTI (9), which is often associated with a programmed cell death called hypersensitive response (HR).

Gram-negative phytopathogenic bacteria use a specialized secretion system, the Hrp type III secretion system (T3SS), to inject effector proteins into host cells (10, 11). To date, many pathogen type III effectors that interfere with plant immunity have been identified. Most of the known targets of these effectors are plant proteins, such as PRRs and their coreceptors, PRR-associated receptor-like cytoplasmic kinases (RLCKs), MAPKs, vesicle transport proteins, and 14-3-3 proteins, involved in the aforementioned disease resistance responses (12, 13). Recent studies have demonstrated enzymatic activities of some type III effectors, including proteases, phosphatases, acetyltransferase, ribosyltransferases, and ubiquitin ligases (13). In *Arabidopsis thaliana*, 165 plant proteins that interact with pathogen effectors have been identified as potential effector targets, and several proteins commonly targeted by multiple pathogen effectors have been shown (14).

On the other hand, only a few effectors that target host cellular molecules other than proteins have been identified. *Phytophthora sojae* Nudix hydrolase effector Avr3b functions as an ADP-ribose/NADH pyrophosphorylase that degrades ADP-ribose and NADH (15) and is considered to act as a negative regulator of plant immunity as AtNUDT7 from *Arabidopsis* (16). *Pseudomonas syringae* pv. *tomato* DC3000 type III effector HopQ1 suppresses PTI by activating the production of cytokinin, probably functioning as a cytokinin-activating enzyme through its nucleoside hydrolase activity (17).

The Hrp T3SS is essential for the pathogenicity of *R. solanacearum* in host plants (18, 19). It is obvious that *R. solanacearum* suppresses PTI through the Hrp T3SS, which enables its proliferation in host plants (20, 21). *R. solanacearum* has been found to possess a large repertoire of 70 to 75 type III effectors (22, 23), which are now called Rips (*Ralstonia* injected proteins) (24). For some Rip effectors, their enzymatic activities inside plant cells have been demonstrated. The *R. solanacearum* RipP2 (PopP2) effector functions as an acetyltransferase that targets WRKY transcription factors (25, 26). Effectors belonging to the RipG (GALA) family function as a pathogen-derived F box protein subunit and are considered to hijack a host SCF E3 ubiquitin ligase complex, although their target proteins have not yet been identified (27). The RipTPS effector functions as a trehalose-6-phosphate synthase (TPS) and produces trehalose-6-phosphate in yeast cells (28). However, the functions of other effector proteins have not yet been clarified in detail.

It has been shown that yeast is a useful model system for the study of the function of effector proteins in eukaryotic cells (29, 30). In this study, we expressed *R. solanacearum* effectors from strain RS1000 (phylotype I, biovar 4) (23) in yeast cells and identified several effectors that inhibit yeast growth. Here, we show that one of these effectors, RipAY (Rip55), functions as a γ-glutamyl cyclotransferase (γ-CTS) that degrades glutathione (GSH), the tripeptide that plays multiple important roles in disease resistance responses in plants (31). We also provide evidence that RipAY is specifically activated by plant cytosolic thioredoxins and degrades GSH in plant cells to suppress PTI. Analysis of an *R. solanacearum* mutant defective in GSH synthesis suggests that the tight regulation of RipAY is required for the effective growth of this bacterium.

## RESULTS

### Identification of *R. solanacearum* type III effector proteins that interfere with yeast growth.

**FIG 1:**
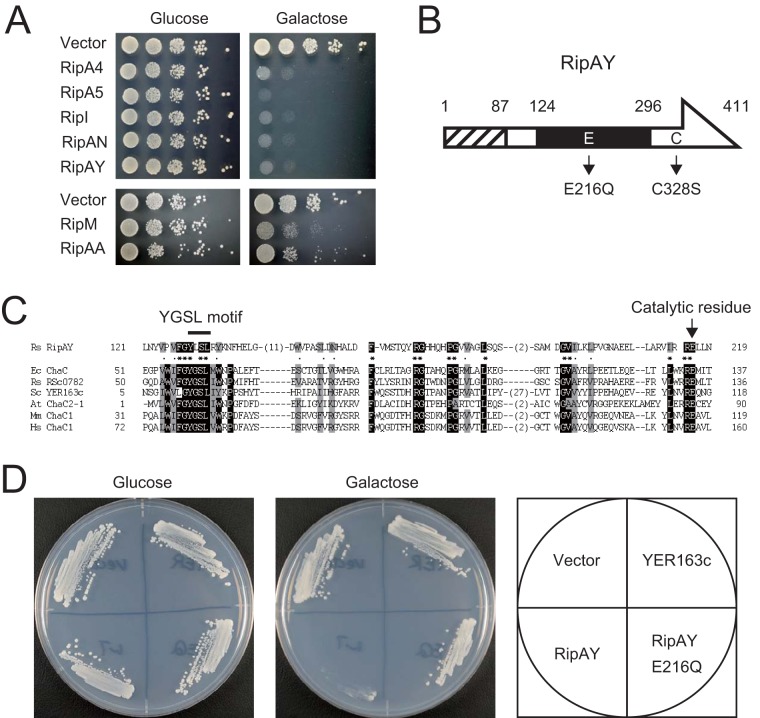
*R. solanacearum* type III effector proteins that interfere with yeast growth. (A) Yeast strains, harboring yeast expression vector pYC2/CT or pYC2/CT carrying the effector genes, were spotted onto repressing medium containing 2% glucose (Glc) or inducing medium containing 2% galactose and 1% raffinose (Gal). Photographs were taken after 2 and 3 days of incubation at 30°C in repressing and inducing media, respectively. (B) Schematic structure of RipAY. Numbers indicate amino acid positions. The hatched box indicates the region required for type III secretion. The solid box indicates the region that shares homology with the ChaC proteins. The putative catalytic glutamic acid residue at position 216 and the sole cysteine residue at position 328 are shown with their mutants. (C) ChaC-like domain of RipAY. The amino acid sequences of the central region of RipAY and the catalytic domain of ChaC family proteins were aligned using the ClustalW software. Conserved amino acids are shaded in black (more than 83% identity) and gray (more than 86% similarity). The signature motif YGSL and the catalytic glutamate residue are shown. *R. solanacearum* possesses another ChaC protein, RSc0782, that shares high homology with *E. coli* ChaC and is considered to be a noneffector. Species abbreviations: Rs, *R. solanacearum*; Ec, *E. coli*; Sc, *S. cerevisiae*; At, *A. thaliana*; Mm, *Mus musculus*; Hs, *Homo sapiens*. (D) Growth of yeast cells expressing RipAY, RipAY E216Q, and *S. cerevisiae* ChaC protein YER163c. Each strain was streaked on the repressing (Glc) and inducing (Gal) media and incubated at 30°C; photographs were taken after 2 and 3 days of incubation, respectively.

To identify RS1000 effector proteins affecting yeast growth, we cloned the entire coding region of each *rip* gene into a low-copy-number yeast plasmid under control of the galactose-inducible *GAL1* promoter. Each plasmid was transformed into the yeast strain YPH499, and the resulting transformant was grown on both repressing and inducing media. We tested the ability of 66 Rips in this yeast assay because six Rips of RS1000, namely, RipJ (Rip22), RipP2-2 (Rip9), RipV1 (Rip12), RipAE (Rip4), RipAT (Rip67), and RipAZ1 (Rip71), have mutations, such as a frameshift mutations and IS insertions, within their genes and were considered to be truncated in RS1000 despite their translocation into plant cells (23). Out of the 66 Rips tested, five effectors, namely, RipA4 (Rip45), RipA5 (Rip56), RipI (Rip1), RipAN (Rip43), and RipAY (Rip55), significantly inhibited yeast growth when expressed in cells (Fig. 1A, upper panel). We also found two effectors, RipM (Rip16) and RipAA (AvrA): the former moderately inhibited yeast growth, and the latter weakly inhibited it (Fig. 1A, lower panel). This result indicates that these seven effectors interfere to various degrees with yeast cellular functions that are required for growth.

Among the five effectors that significantly inhibited yeast growth, we focused on the RipAY effector because a homology search of databases using the BLASTP algorithm revealed that it contains a putative ChaC domain in its central region (Fig. 1B). The ChaC family proteins are γ-glutamyl cyclotransferases (γ-GCTs), acting specifically to degrade glutathione (GSH) (32, 33). Putative active site residues (the signature motif YGSL and catalytic glutamate residue) and the surrounding amino acids conserved in the ChaC family proteins were also conserved in RipAY (Fig. 1C). It has been reported that mutations in the catalytic glutamic acid residue completely abolish the γ-GCT activity of ChaC family proteins (32, 33). To examine whether the putative ChaC domain of RipAY is required for its function, we changed the essential glutamic acid at position 216 to glutamine and constructed the putative catalytically inactive mutant RipAY E216Q (Fig. 1B). We found that RipAY E216Q no longer inhibits yeast growth when expressed in cells (Fig. 1D). This finding indicates that the ChaC domain is essential for the function of RipAY. To examine whether the *Saccharomyces cerevisiae* ChaC homologue YER163c, which has been shown to exhibit γ-GCT activity (32), also inhibits yeast growth, we constructed a yeast strain expressing YER163c. However, the expression of YER163c did not inhibit yeast growth (Fig. 1D).

### RipAY expression induces GSH depletion in yeast cells.

**FIG 2:**
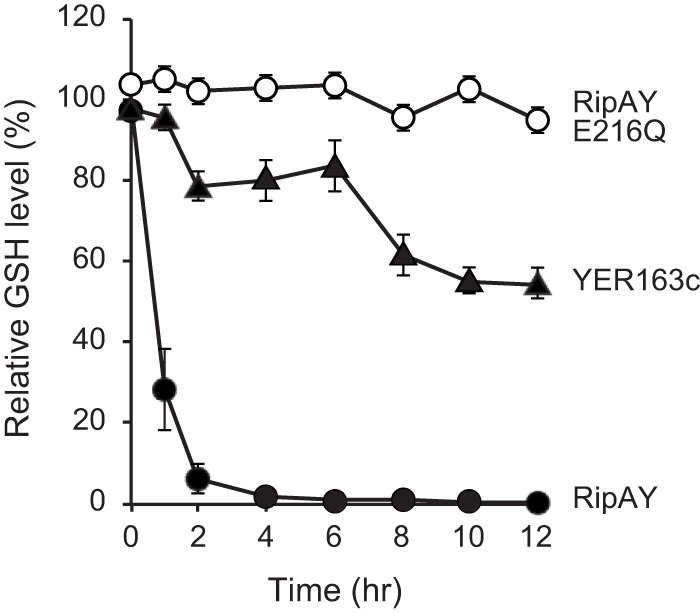
The expression of RipAY, but not RipAY E216Q, induces GSH depletion in yeast cells. The GSH level in yeast cells expressing RipAY (solid circles), RipAY E216Q (open circles), and YER163c (solid triangles) was measured every 2 h after induction. The relative GSH levels (in percentages) are shown in comparison with the vector control. The data are means ± standard deviations (SD) from three independent experiments.

Although the expression of YER163c did not affect yeast growth, the important role of putative catalytic glutamate residue (Glu-216) of RipAY in yeast growth inhibition suggested that RipAY functions as γ-GCT and degrades glutathione in yeast. Therefore, we next monitored GSH levels in yeast cells that induced the expression of RipAY over the time course given. Interestingly, the GSH level in yeast cells rapidly decreased immediately after the expression of RipAY was induced, and it decreased to less than 1% compared with that of control yeast cells harboring the empty vector 6 h after induction (Fig. 2). In contrast, such a rapid decrease in GSH level was not observed in yeast cells expressing RipAY E216Q. This finding clearly indicates that RipAY depletes the GSH pool in yeast cells even under ongoing GSH synthesis, probably inhibiting yeast growth on the synthetic minimal medium. On the other hand, the GSH level in yeast cells expressing YER163c decreased gradually and reached the minimum 10 to 12 h after induction, at which point, the GSH level decreased to 50 to 60% of that in control cells. It has also been reported that yeast and mammalian ChaC1 proteins decreased the GSH level to 50 to 60% when expressed in yeast cells (32). These findings suggest that the RipAY effector has an extremely high GSH degradation activity compared with already known ChaC family proteins. We also monitored GSH levels in yeast cells that induced the expression of RipA4, RipA5, RipI, and RipAN. Although these four effectors significantly inhibited yeast growth when expressed in yeast cells, none of these effectors showed an ability to induce GSH depletion in yeast cells (see Fig. S1 in the supplemental material).

### The GSH degradation activity of RipAY is activated by yeast and plant cell extracts.

**FIG 3:**
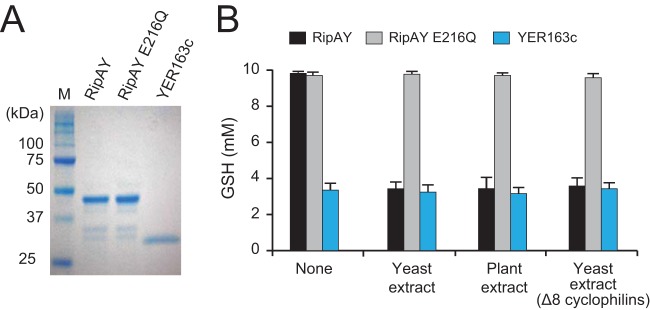
The GSH degradation activity of RipAY is activated by unidentified eukaryotic factors. (A) Purified recombinant proteins of N-terminal His-tagged RipAY, RipAY E216Q, and YER163c. (B) GSH degradation assay. GSH (10 mM) was incubated with 3 µg of the recombinant ChaC protein for 60 min at 30°C in 100 µl of the 50 mM Tris-HCl (pH 8.0) reaction mixture. Yeast and plant extracts and *E. coli* lysates were added to the reaction mixture as described in Materials and Methods. The sample was then boiled at 95°C for 5 min to stop the reaction, and the GSH level was measured. The data are means ± SD from three independent experiments.

To clarify the GSH degradation activity of RipAY *in vitro*, we expressed and purified the recombinant protein of RipAY, RipAY E216Q, and YER163c using its N-terminal His tag in *E. coli* (Fig. 3A). When the yeast ChaC protein YER163c was incubated with GSH, the GSH level in the reaction mixture decreased (Fig. 3B), indicating that YER163c degrades GSH *in vitro* as previously reported (32). However, no decrease in GSH level was observed in the reaction mixture with RipAY (Fig. 3B). Because the expression of N-terminal His-tagged RipAY inhibited yeast growth (see Fig. S2 in the supplemental material), the recombinant RipAY was considered to maintain the GSH degradation activity. Although we tested various reaction conditions, no GSH degradation activity of RipAY was detected *in vitro*. A few bacterial virulence proteins have been found to require host eukaryotic proteins in their enzymatic activities (34, 35); hence, we attempted to add an extract of yeast cells to the reaction mixture. Interestingly, RipAY degrades GSH in the presence of the yeast extract (Fig. 3B). We also added an extract of *Arabidopsis* leaves to the reaction mixture and found that RipAY degrades GSH in the presence of the plant extract. The putative catalytically inactive RipAY E216Q protein did not degrade GSH even in the presence of yeast and plant extracts. It has been shown that the protease activity of the *Pseudomonas syringae* type III effector AvrRpt2 is specifically activated by eukaryotic cyclophilins from yeast and plants (35). We therefore added an extract of yeast cells lacking all the eight cyclophilins, which could not activate AvrRpt2, to the reaction mixture and found that RipAY can degrade GSH in the absence of cyclophilins (Fig. 3B). These findings indicate that the GSH degradation activity of RipAY is activated by as-yet-unidentified eukaryotic factors commonly existing in yeast and plants other than cyclophilins.

### Identification of yeast proteins required for the GSH degradation activity of RipAY.

**FIG 4:**
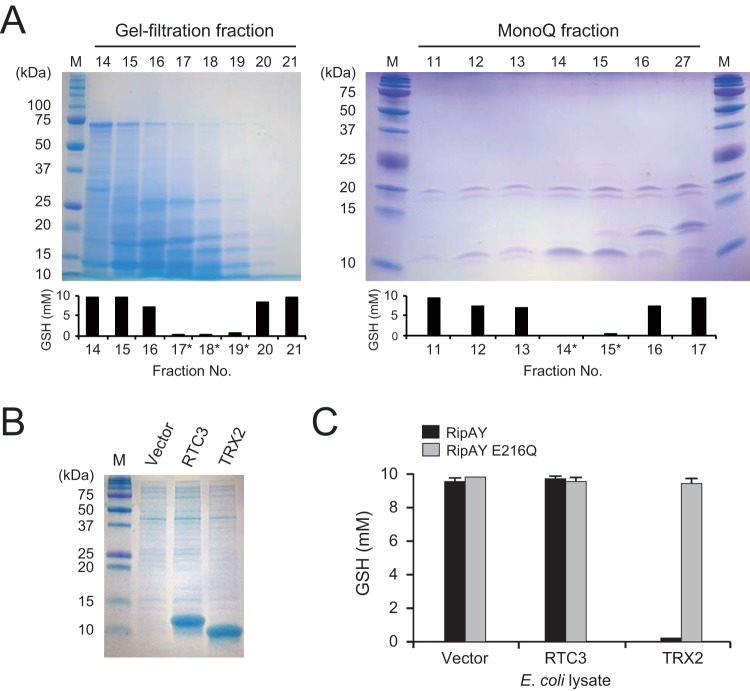
Purification and identification of yeast protein that activate RipAY. (A) Purification of RipAY activating protein from yeast cell extract by gel-filtration chromatography (left) and anion-exchange chromatography (right). After the dialysis of each 1-ml fraction, 1/10 vol of a sample was added to the reaction mixture containing 3 µg RipAY and 10 mM GSH substrate, and the mixture was incubated for 60 min at 30°C. Fractions surrounding the fraction showing RipAY activation were analyzed by SDS-PAGE (top panel). Fractions analyzed further are indicated by asterisks. The mean GSH level of three independent experiments is shown (bottom graph). (B) Lysates used for the RipAY activation assay. Lysates of *E. coli* strains expressing RTC3 and TRX2 were analyzed by SDS-PAGE. (C) Activation of RipAY by recombinant yeast protein. Each *E. coli* lysate containing the recombinant yeast RTC3 or TRX2 protein was added to the reaction mixture and assayed for its capability to activate RipAY.

To characterize the eukaryotic factor that activates the GSH degradation activity of RipAY, we first heat treated yeast lysate at 95°C for 20 min and found that the heat-treated extract retained its capability to activate RipAY (data not shown). Next, using membrane filters, we dialyzed the heat-treated yeast extract into several fractions. A flowthrough fraction from a membrane filter having a cutoff molecular mass of 100 kDa, but not that from a 10-kDa-cutoff membrane filter, showed a capability to activate the GSH degradation activity of RipAY (data not shown), suggesting that the activation factor has a protein with a molecular mass in the range of 10 to 100 kDa. Therefore, yeast proteins having such a molecular mass were further fractioned by gel filtration chromatography, and the obtained fractions were assayed for their capability to activate the GSH degradation activity of RipAY (Fig. 4A). Proteins from RipAY activation-positive fractions (gel filtration fractions 17, 18, and 19) were further fractioned by anion-exchange chromatography. Finally, an 11-kDa protein band correlated with the GSH degradation activity was identified (Mono Q fractions 14 and 15). Mass spectrometry (MS) revealed that this protein band mainly includes two yeast proteins, RTC3 (YHR087W) and thioredoxin TRX2 (see Fig. S3 in the supplemental material). To examine which protein is the true activator of RipAY, we expressed their recombinant proteins in *E. coli* (Fig. 4B). When the lysate of *E. coli* cells expressing each recombinant protein was added to the reaction mixture, the lysate containing TRX2, but not that containing RTC3, markedly activated RipAY to degrade GSH (Fig. 4C). The putative catalytically inactive RipAY E216Q showed no GSH degradation activity in the presence of TRX2. These results indicate that TRX2 is a eukaryotic activator of RipAY in yeast cells.

### Purified recombinant yeast thioredoxin, but not *E. coli* thioredoxin, activates the GSH degradation activity of RipAY.

**FIG 5:**
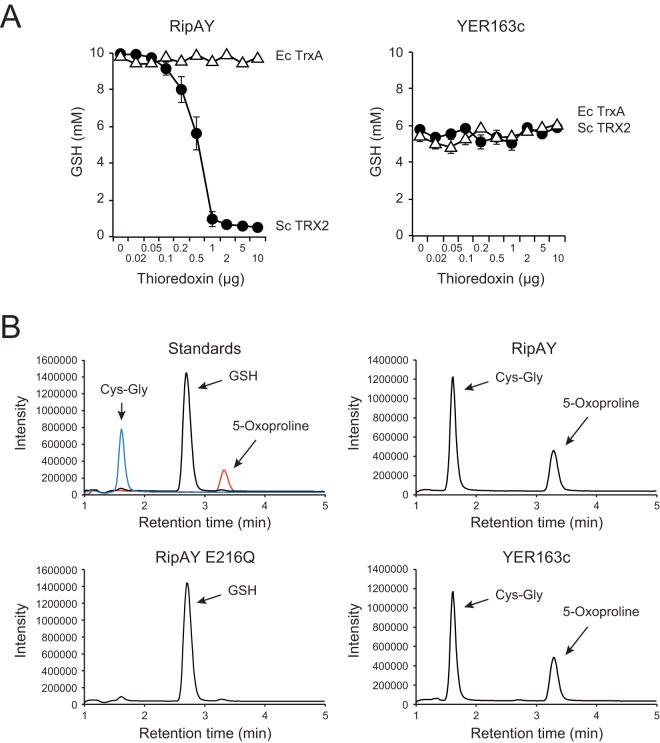
Activation of the GSH degradation activity of RipAY by purified recombinant thioredoxin. (A) Effect of purified recombinant thioredoxin on the GSH degradation activity of RipAY and YER163c. GSH (10 mM) was incubated with 1 µg of RipAY (left) and 2 µg of YER163c (right) in the presence of various amounts of yeast TRX2 (solid circles) and *E. coli* TrxA (open triangles) thioredoxins for 60 min at 30°C in 100 µl of 50 mM Tris-HCl (pH 8.0) reaction mixture. (B) RipAY exhibits γ-glutamyl cyclotransferase activity. GSH (1 mM) was incubated with 3 µg of RipAY (upper right panel), RipAY E216Q (bottom left panel), and YER163c (bottom right panel) for 60 min at 30°C in 100 µl of 50 mM Tris-HCl (pH 8.0) reaction mixture. For RipAY and RipAY E216Q, 2 µg of yeast TRX2 thioredoxin was added to the reaction mixture. The reaction mixture was then incubated at 95°C for 5 min to stop the reaction, and the substrate and the degradation products in the terminated sample were analyzed by LC-MS as described in Materials and Methods. The substrate and products in the reaction mixture were identified with authentic standards (upper left panel, with the individually detected three peaks shown together). GSH (*m*/*z* =308.3), 5-oxoproline (*m*/*z* = 130.1), and Cys-Gly (*m*/*z* =179.2) were detected at 2.71, 3.32, and 1.63 min, respectively.

To confirm the ability of TRX2 to activate the GSH degradation activity of RipAY, we added the purified recombinant protein of TRX2 that has the N-terminal His tag to the reaction mixture. As a result, the GSH degradation activity of RipAY markedly increased with the amount of the TRX2 protein (Fig. 5A). On the other hand, TRX2 did not change the GSH degradation activity of the yeast ChaC protein YER163c. We evaluated the GSH degradation activities of fully activated RipAY (80 µmol GSH degradation/min/mg protein) and YER163c (3.7 µmol GSH degradation/min/mg protein). Considered the molecular masses of RipAY (44.2 kDa) and YER163c (26.3 kDa), as expected, the fully activated RipAY showed 30- to ~40-fold-higher activity than YER163c at the enzyme level.

Thioredoxins are small proteins (approximately 12 kDa) involved in cell redox regulation and present in all organisms from prokaryotes to higher eukaryotes (36). However, the GSH degradation activity of RipAY was not activated by *E. coli* lysates that include bacterial thioredoxins (Fig. 4C). To determine whether bacterial thioredoxin activates the GSH degradation activity of RipAY, we added the purified recombinant protein of *E. coli* thioredoxin TrxA that has the N-terminal His tag to the reaction mixture. Interestingly, TrxA showed no ability to activate RipAY, despite the addition of an excess amount of the protein (Fig. 5A). These findings clearly show that RipAY requires yeast TRX2, but not *E. coli* TrxA, for its GSH degradation activity.

To confirm the γ-GCT activity of RipAY, we analyzed the products of the reaction mixture by liquid chromatography-mass spectrometry (LC-MS) as described in Materials and Methods. When RipAY was incubated with GSH in the presence of TRX2, the peak corresponding to GSH disappeared, and new peaks corresponding to 5-oxoproline and Cys-Gly appeared as well as the products of the control reaction using YER163c (Fig. 5B). The generation of 5-oxoproline and Cys-Gly from GSH demonstrated the γ-GCT activity of RipAY.

### Plant cytosolic thioredoxins activate the GSH degradation activity of RipAY in a disulfide reductase activity-independent manner.

**FIG 6:**
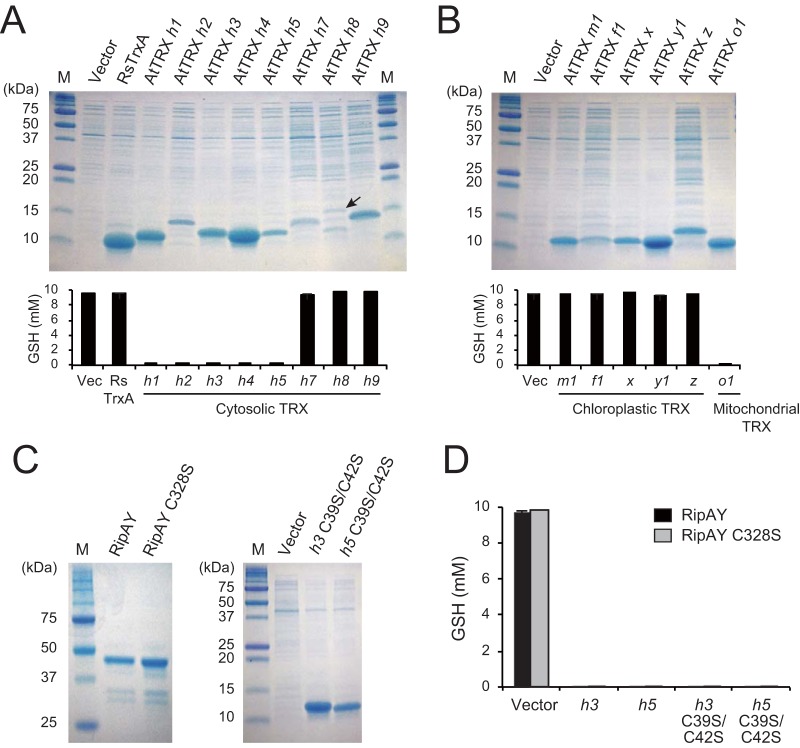
Plant cytosolic thioredoxins in a redox-independent manner. (A) Effect of plant cytosolic thioredoxins on the activation of RipAY. Each *E. coli* lysate containing *Arabidopsis* thioredoxin (top panel) was incubated with 3 µg of RipAY and 10 mM GSH for 60 min at 30°C in 100 µl of 50 mM Tris-HCl (pH 8.0) reaction mixture. The mean GSH level from three independent experiments is shown (bottom graph). Vec, empty vector. (B) Effect of chloroplastic and mitochondrial thioredoxins. (C) Purified recombinant RipAY and RipAY C328S, and *E. coli* lysates used for the RipAY activation assay. Lysates containing recombinant AtTRX *h3* C39S C42S and AtTRX *h5* C39S C42S were analyzed by SDS-PAGE. (D) Each of the AtTRXs *h3* and *h5* and each of their mutants defective in disulfide reductase activity was incubated with 3 µg of RipAY and RipAY C328S and 10 mM GSH for 60 min at 30°C in 100 µl of 50 mM Tris-HCl (pH 8.0) reaction mixture. The mean GSH level from three independent experiments is shown.

The Rip effector proteins of *R. solanacearum* are considered to exert their virulence functions inside the plant cell. The GSH degradation activity of RipAY is activated by the extract from *Arabidopsis* leaves (Fig. 3B), suggesting that RipAY is enzymatically inactive in *R. solanacearum* but activated by plant thioredoxins after its translocation into host cells. We added the lysate of *E. coli* cells expressing *R. solanacearum* TrxA thioredoxin and confirmed that the pathogen thioredoxin also showed no ability to activate RipAY. In *Arabidopsis*, at least 20 thioredoxins have been identified and classified into seven major types: *f*, *m*, *x*, *y*, *z*, *o*, and *h* (37, 38). To determine whether RipAY is activated by plant thioredoxins, we first constructed *E. coli* strains expressing the eight *Arabidopsis* cytosolic *h*-type thioredoxins. When the lysate of each strain was added to the reaction mixture, five thioredoxins, namely, AtTRXs *h1*, *h2*, *h3*, *h4*, and *h5*, activated the GSH degradation activity of RipAY (Fig. 6A). The remaining AtTRXs, the *h7*, *h8*, and *h9* thioredoxins, showed no ability to activate RipAY. We next tested the effect of plant chloroplastic and mitochondrial thioredoxins on the activation of RipAY. In contrast to the cytosolic thioredoxins, none of the *Arabidopsis* chloroplastic *m*-, *f*-, *x*-, *y*- and *z*-type thioredoxins activated the GSH degradation activity of RipAY (Fig. 6B). On the other hand, *Arabidopsis* mitochondrial *o*-type thioredoxin AtTRX *o1* activated RipAY. These results indicate that RipAY is activated by plant cytosolic and mitochondrial thioredoxins in plant cells.

Thioredoxins are known to regulate the activity of target proteins by reduction of the intramolecular disulfide bridges formed within a protein or intermolecular disulfide bridges formed between proteins (39). RipAY possesses only one cysteine (Cys) residue in its C-terminal region (Fig. 1B), leading to the hypothesis that RipAY forms an inactive homodimer by forming the intermolecular disulfide bridge through this Cys residue. In such a case, plant thioredoxins might reduce the disulfide bridge, producing an active monomer that can degrade GSH. To test this hypothesis, we constructed RipAY C328S, in which the cysteine of position 328 was changed to serine (Fig. 1B). The recombinant protein of RipAY C328S was expressed in *E. coli* and purified using its N-terminal His tag (Fig. 6C). However, RipAY C328S exhibited no GSH degradation activity without adding plant cytosolic thioredoxins (Fig. 6D). This finding suggested that the GSH degradation activity of RipAY is regulated in a disulfide-bridge-independent manner. To confirm whether the disulfide reductase activity of thioredoxin is unnecessary for the activation of RipAY, we constructed two thioredoxin mutants, AtTRX *h3* C39S C42S and AtTRX *h5* C39S C42S, in which two essential cysteine residues at positions 39 and 42 in the active site were changed to serine (Fig. 6C). Both AtTRX *h3* C39S C42S and AtTRX *h5* C39S C42S thioredoxins maintained the ability to activate the GSH degradation activity of RipAY (Fig. 6D). These results clearly show that the disulfide reductase activity of plant thioredoxin is not required for the activation of RipAY.

### RipAY expression induces GSH depletion in plant cells and suppresses plant immunity.

**FIG 7:**
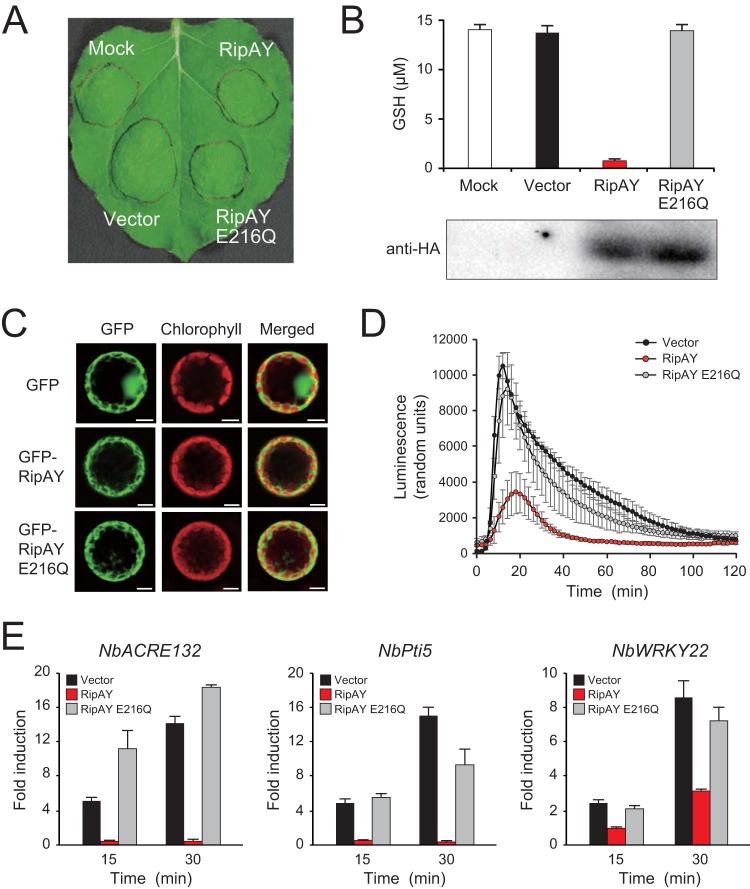
Effect of *Agrobacterium*-mediated transient expressions of RipAY and RipAY E216Q in *N. benthamiana*. (A) Leaves were infiltrated with *A. tumefaciens* strain GV3101 harboring the binary vector carrying the effector genes or empty vector. “Mock” represents the infiltration of buffer only. Photographs were taken 2 days after agroinfiltration. (B) GSH level in the infiltrated leaves. The GSH levels were measured 2 days after infiltration. The data are means ± SD from three independent experiments. (C) Subcellular localization of RipAY. GFP, GFP-RipAY, and GFP-RipAY E216Q were each transiently expressed in leaves by agroinfiltration. Two days after infiltration, protoplasts were prepared from infiltrated leaves, and the subcellular localization of GFP fluorescence was monitored by a confocal laser scanning microscopy. (D) ROS production in the infiltrated leaves. Two days after infiltration, leaf disks were treated with the flg22 elicitor at 100 µM, and the flg22-triggered ROS production was measured as photon counts for 120 min. The data are means ± SD from eight independent leaf disks. (E) Expression of defense-related genes in the infiltrated leaves. Total RNAs were extracted from leaves treated with flg22 at the indicated times in minutes, and the expression of PTI marker genes were analyzed by RT-qPCR.

To clarify the GSH degradation activity of RipAY in plant cells, we transiently expressed the N-terminal hemagglutinin (HA)-tagged RipAY and RipAY E216Q in *Nicotiana benthamiana* leaves using an *Agrobacterium*-mediated transient expression system (agroinfiltration). The expression of RipAY induced no visible cell death in leaves 48 h after agroinfiltration (Fig. 7A). As expected, the expression of RipAY, but not that of RipAY E216Q, decreased the GSH levels of plant leaves to less than 5% of that of the vector control 2 days after agroinfiltration (Fig. 7B). We confirmed by Western blot analysis using an anti-HA antibody that both the RipAY and RipAY E216Q proteins were expressed in plant leaves. This finding clearly shows that RipAY degrades GSH in plant cells.

We next examined the subcellular localization of RipAY with green fluorescent protein (GFP) fusions. The GFP, GFP-RipAY, and GFP-RipAY E216Q proteins were transiently expressed in *N. benthamiana* leaves, and protoplasts were prepared from leaves 2 days after agroinfiltration. The GFP fluorescence signal of GFP alone localized to the cytoplasm and nucleus, whereas those of GFP-RipAY and GFP-RipAY E216Q localized to the cytoplasm (Fig. 7C). This observation suggests that RipAY functions in plant cytoplasm.

GSH plays multiple important roles in plant disease resistance responses (31). To test whether RipAY shows an ability to suppress plant PTI responses, we treated *N. benthamiana* leaf disks transiently expressing effectors with flg22, a PAMP elicitor of the conserved 22-amino-acid motif of bacterial flagellin. We observed that the expression of RipAY, but not that of RipAY E216Q, markedly decreased ROS production level induced by 100 nM flg22 (Fig. 7C). We also monitored the expression of several PTI marker genes in *N. benthamiana*, namely, *NbACRE132*, *NbPti5*, and *NbWRKY22*: expression was highly induced by flg22 treatment (40–42). Interestingly, the expression of RipAY, but not RipAY E216Q, significantly reduced the flg22-triggered expression of PTI marker genes (Fig. 7D). These findings show that RipAY suppresses plant PTI responses through its GSH degradation activity.

### Mutations in the *gshA* and *gshB* genes of *R. solanacearum* affect tolerance to stress conditions and growth.

The inactivation of the GSH degradation activity of RipAY in *R. solanacearum* cells suggests that GSH plays an important role in this pathogen. In bacteria, GSH is synthesized by two steps catalyzed by γ-glutamylcysteine synthetase and glutathione synthase, encoded by the *gshA* and *gshB* genes, respectively (43). To examine the contribution of GSH to the growth of this pathogen under stress conditions, we constructed a Δ*gshAB* mutant of *R. solanacearum*. The Δ*gshAB* mutant was grown in a nutrient-rich medium and then shifted to the minimal medium after a short time, and the GSH levels of bacterial cells were measured and compared with those of the wild type. As expected, GSH was not detectable in the Δ*gshAB* mutant under our experimental conditions (Table 1). To determine the role of GSH in the tolerance of *R. solanacearum* to oxidative and toxic stresses, we performed disk diffusion sensitivity tests. The *R. solanacearum* Δ*gshAB* mutant formed an obviously large growth inhibition zone in the presence of H_2_O_2_ and methylglyoxal compared with the wild type (Table 1). This result indicates that GSH is involved in the tolerance of *R. solanacearum* to oxidative and toxic stresses.

**TABLE 1:** GSH level of *Ralstonia solanacearum* strains and stress sensitivity test

Strain	Relevant genotype	Relative GSH level (μmol/μg)^a^	Radius of growth inhibition zone (mm)^b^	
			H_2_O_2_	Methylglyoxal
RS1002	*gshA*^+^ *gshB*^+^	1.03 ± 0.08	7.7 ± 0.5	10.3 ± 0.6
RS1701	Δ*gshAB*::Sm^r^/Spc^r^	ND^c^	11.0 ± 0.5	14.0 ± 0.4

a GSH level (micromoles of GSH per microgram of total soluble protein) was measured in cell extracts from *R. solanacearum* strains prepared as described in Materials and Methods. The values are means ± SD from three independent experiments.

b The mean radius of the growth inhibition zones was measured as described in Materials and Methods. The values are means ± SD from five independent experiments.

c ND, not detected.

**FIG 8:**
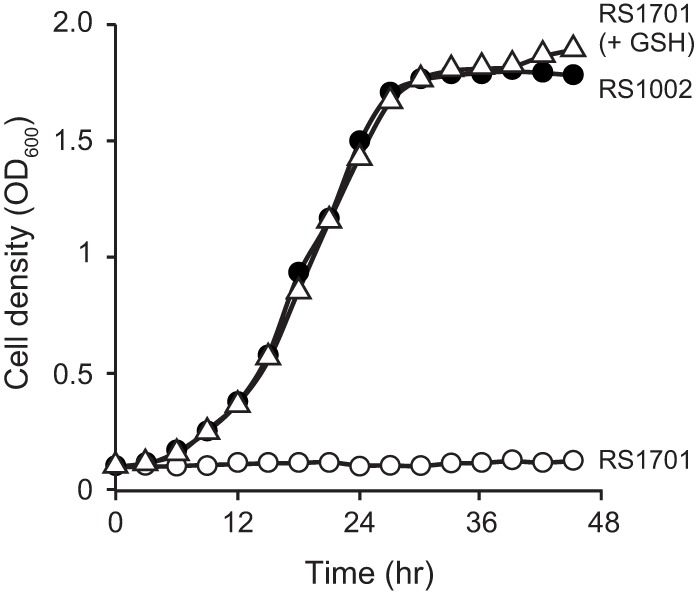
Effect of GSH on the growth of *R. solanacearum* in minimal medium. *R. solanacearum* strains RS1002 (wild type [solid circles]) and RS1701 (Δ*gshAB* [open circles]) were diluted in 1/4 M63 glucose minimal medium to an OD_600_ of 0.1 as described in Materials and Methods. The cultures were incubated at 28°C, and their cell densities were measured over the time course given. The growth of RS1701 was also measured with 1/4 M63 glucose minimal medium supplemented with 1 mM GSH (open triangles). The data are means from three independent experiments.

To examine the role of GSH in the growth of *R. solanacearum*, we monitored the growth of *R. solanacearum* strains in minimal medium. Growth of the Δ*gshAB* mutant was severely affected in the minimal medium compared with that of the wild type (Fig. 8). The growth arrest of the Δ*gshAB* mutant was fully restored by the addition of 1 mM GSH to the minimal medium. This finding shows that GSH is essential for the growth of *R. solanacearum*.

## DISCUSSION

In this study, we demonstrated that *R. solanacearum* type III effector RipAY is a GSH-degrading enzyme. We obtained several lines of evidence. (i) RipAY was found to share homology with the ChaC family proteins that function as γ-GCTs to specifically degrade GSH (32). Although the entire homology is low, the YGSL motif and catalytic glutamic acid residue that are important for γ-GCT activity are conserved with their surrounding amino acids (Fig. 1C). (ii) The expression of RipAY, but not catalytically inactive RipAY E216Q, induced rapid GSH depletion in yeast (Fig. 2). (iii) The recombinant RipAY, but not RipAY E216Q, degrades GSH into 5-oxoproline and Cys-Gly in the presence of eukaryotic thioredoxins *in vitro* (Fig. 5 and 6). On the basis of these findings, we conclude that RipAY is a new ChaC family protein that requires eukaryotic thioredoxins to degrade GSH. As far as we know, RipAY is the first effector protein identified that directly targets GSH.

The *in vitro* GSH degradation activity of fully activated RipAY is very high compared with that of the yeast ChaC protein YER163c (Fig. 5). The expression of RipAY but not YER163c inhibited yeast growth (Fig. 1D). Yeast mutants defective in GSH synthesis show GSH auxotrophy in minimal medium (44). Therefore, it is considered that the growth defect of yeast cells by RipAY is caused by GSH depletion. The observation that the expression of catalytically inactive RipAY E216Q in yeast cells induced neither growth defect (Fig. 1D) nor the GSH depletion (Fig. 2) well supports this idea. The expression of YER163c decreased the GSH level in yeast cells to 50 to ~60% of that in the wild type. Because YER163c functions in the γ-glutamyl cycle to maintain GSH homeostasis in yeast cells, the GSH degradation activity might be low.

The high GSH degradation activity of RipAY contributes to the depletion of the GSH pool in plant cells (Fig. 7B), in which the concentration is maintained at 2 to 3 mM (45, 46). GSH plays important roles in disease resistance responses of plants. The *Arabidopsis* phytoalexin-deficient2-1 (*pad2-1*) mutation is a mutant allele of *GSH1* encoding γ-glutamylcysteine synthetase (GSH1), the first enzyme in GSH synthesis (47). The *pad2-1* mutant, which contains only ~20% of the GSH level compared with the wild type (47), shows multiple defects in disease resistance responses under biotic stresses: (i) lower plasma membrane depolarization and low levels of production of H_2_O_2_ and salicylic acid (SA) (31), as well as (ii) low accumulation levels of phytoalexins, such as camalexin and indole glucosinolates (48, 49). As in the *pad2-1* mutant, the expression of RipAY markedly decreased the flg22-triggered ROS production and the subsequent expression of PTI marker genes in *N. benthamiana* (Fig. 7C and D), indicating that RipAY suppresses plant PTI responses through GSH degradation. The effect of RipAY on other plant immunity responses, such as ETI, should be clarified in future work.

The *pad2-1* mutants showed a higher susceptibility to virulent bacterial pathogens than the wild type (48). It is considered that *R. solanacearum* uses RipAY to decrease the GSH level in plant cells and increase susceptibility in the early stage of infection. *R. solanacearum* Δ*ripAY* mutants showed no marked difference in multiplication in host eggplants compared with the wild type (see Fig. S4 in the supplemental material). However, this is not surprising because pathogen effectors may have a functional redundancy, and a single mutation in effector genes has no effect on bacterial virulence in many cases. Although we were unable to find no effectors other than RipAY that induce GSH depletion in yeast cells (see Fig. S1 in the supplemental material), *R. solanacearum* might possess as-yet-unidentified effectors that degrade GSH only in plant cells. From another viewpoint, it is considered that *R. solanacearum* possesses effectors other than RipAY that suppress plant immunity to enable the effective growth of the *ripAY* mutant in host plants. Nevertheless, we believe that RipAY plays an important contribution during pathogenesis because RipAY is a core type III effector that is conserved in all of the *R. solanacearum* species complex genomes sequenced (24).

The GSH degradation activity of RipAY was not activated by bacterial thioredoxins from *E. coli* and *R. solanacearum* (Fig. 5 and 6), indicating that RipAY is inactive in bacterial cells. In many Gram-negative bacteria, GSH plays important roles in tolerance to abiotic stresses, such as reactive oxygen species and toxic compounds, by maintaining the redox homeostasis in bacterial cells and the detoxification of toxic compounds (43). In some *Salmonella enterica* strains, the deficiency in GSH synthesis leads to GSH auxotrophy in minimal medium even under nonstress conditions (50). *Sinorhizobium meliloti* strains lacking GSH are unable to grow in minimal medium and lose the ability to develop symbiotic interactions in plants (51). *R. solanacearum* strains lacking GSH also show increased sensitivities to H_2_O_2_ and methylglyoxal and GSH auxotrophy in minimal medium (Table 1; Fig. 8). To prevent these functional deficiencies in bacterial cells by GSH degradation, RipAY may be expressed as an inactive form in *R. solanacearum* cells.

The inactive RipAY effector is activated by plant thioredoxins. Interestingly, RipAY is activated by plant cytosolic *h*-type thioredoxins but not by the chloroplastic *m*-, *f*-, *x*-, *y*-, and *z*-type thioredoxins from *A. thaliana* (Fig. 6). In plants, the synthesis of γ-glutamylcysteine, the GSH precursor, is exclusively promoted by GSH1 in the plastid, whereas the synthesis of GSH is promoted by glutathione synthetase (GSH2) in both the cytosol and plastids (52). It has been shown that the GSH pool level in the plant cytosol rather than that in the plastid is important for the appropriate activation of defense responses (53). The ChaC family proteins are considered to degrade cytosolic GSH to maintain GSH homeostasis (32, 33). Therefore, it is reasonable to consider that RipAY is activated by plant *h*-type thioredoxins in the plant cytosol. The subcellular localization analysis using GFP fusion indicated that RipAY localizes to the plant cytoplasm (Fig. 7C), well supporting this idea.

In our *in vitro* assay, the GSH degradation activity of RipAY was activated by five of eight *Arabidopsis h*-type thioredoxins, namely, AtTRXs *h1*, *h2*, *h3*, *h4*, and *h5*, but not by the other three *h*-type thioredoxins, AtTRXs *h7*, *h8*, and *h*9 (Fig. 6A). It is noteworthy that the expression of genes encoding the former five thioredoxins was detected in almost all plant organs at various levels, whereas the levels of expression of genes encoding AtTRXs *h7* and *h8* were very low, and expression was detectable in limited organs (54). Although the expression of AtTRX *h9* was detectable in all plant organs, AtTRX *h*9 was localized in the plasma membrane of plant cells (55). It is interesting that the preferences for thioredoxins by RipAY are fine-tuned to those expressed in the plant cytosol at high levels but not those expressed at quite low levels. Among the aforementioned five *h*-type thioredoxins, AtTRXs *h3* and *h5* are highly expressed in *Arabidopsis*: the former is constitutively expressed at high levels, and the latter is highly induced upon pathogen infection (54). Therefore, TRXs *h3* and *h5* are potential activators of RipAY by interacting with RipAY, which changes to its active form.

RipAY was also activated by a mitochondrial *o*-type thioredoxin (Fig. 6B). Sequence comparisons suggest that thioredoxins *m*, *x*, *y*, and *z* are related to prokaryotic thioredoxins, whereas thioredoxins *f*, *h*, and *o* are specifically of eukaryotic origins (37, 38, 56). For this reason, the activation of RipAY might be adapted to mitochondrial *o*-type thioredoxin. It is obvious that the activation of RipAY is determined by a specific amino acid domain or structure of plant thioredoxins. It is necessary to determine such an activation domain in thioredoxin sequences.

The mechanism underlying the activation of the GSH degradation activity of RipAY by thioredoxin remains unclear in this article. We showed that the disulfide reductase activity of plant *h*-type thioredoxin is not required for the activation of RipAY (Fig. 6D). This finding suggests that plant *h*-type thioredoxins activate RipAY through their interaction with specific protein domains other than the two active-site Cys residues. This idea well explains the observation that bacterial thioredoxins showing disulfide reductase activity did not activate RipAY (Fig. 5 and 6). In animal cells, thioredoxin has been found to directly bind to apoptosis signal-regulating kinase (ASK1) with a domain other than the two active-site Cys residues and inhibited the kinase as a direct inhibitor (57). Although plant *h*-type thioredoxins act as activators, the direct interaction between RipAY and *h*-type thioredoxins should be confirmed in future work.

**FIG 9:**
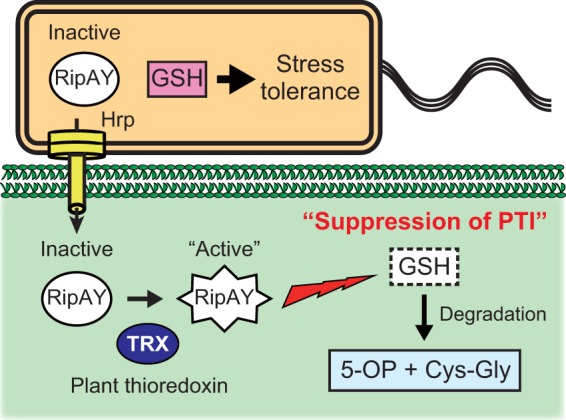
Model of the role of RipAY in the plant-*R. solanacearum* interaction. *R. solanacearum* injects the RipAY effector into host plant cells via the Hrp type III secretion system in the early stage of infection. RipAY is activated by plant cytosolic thioredoxins and degrades GSH, resulting in the suppression of plant PTI responses. On the other hand, RipAY is maintained as an inactive form and does not degrade GSH in bacterial cells, enabling *R. solanacearum* to protect itself from various oxidative and toxic stresses in plants through its GSH-mediated stress tolerance systems. 5-OP, 5-oxoproline.

It has been considered that the enzyme activity of effectors that target substrates that commonly exist in cells of a pathogen and its host is tightly regulated because the activity of protease effectors, such as YopJ, AvrBsT, and AvrXv4, cannot be detected *in vitro* (58). The first answer is that eukaryotic cyclophilin ROC1 activates the protease activity of the *P. syringae* cysteine protease effector AvrRpt2 (35). A eukaryotic thioredoxin is a novel host protein that regulates the enzymatic activity of effector as an activator (Fig. 9). It will be interesting to determine whether such a regulatory domain “thioredoxin switch” exists in other effectors and controls their enzyme activities.

## MATERIALS AND METHODS

### Bacterial and yeast strains and media.

The bacterial and yeast strains used in this study are listed in Table S1 in the supplemental material. *R. solanacearum* RS1002 is a spontaneous Nal^r^ derivative of the wild-type strain RS1000 (phylotype I, biovar 4, isolated from tomato in Japan) (19). *S. cerevisiae* KDY97.1a (59) was used to prepare yeast lysates lacking all the eight cyclophilins. The growth conditions, media, and antibiotics used for the *R. solanacearum*, *E. coli*, and *Agrobacterium tumefaciens* strains were described previously (19, 60). The yeast strains were grown at 30°C in YPAD medium (yeast extract-peptone-dextrose medium with adenine added) or in synthetic complete medium lacking uracil (SC −Ura medium) and supplemented with 2% glucose for repression medium or 2% galactose and 1% raffinose for inducing medium (61).

### Construction of yeast expression plasmids.

The entire coding region of each *rip* was PCR amplified using Pwo SuperYield DNA polymerase (Roche, Mannheim, Germany) from genomic DNA of *R. solanacearum* RS1000 and inserted into the low-copy-number yeast expression vector pYC2/CT (Invitrogen, Carlsbad, CA) under control of the galactose-inducible *GAL1* promoter. The primer sets used for the construction of gene cassettes are listed in Table S2 in the supplemental material.

### Expression and purification of recombinant proteins in *E. coli*.

For the production of N-terminal His-tagged recombinant proteins, the entire coding region of each target gene was PCR amplified from genomic DNA from bacteria, yeast, and plants, using the primer sets listed in Table S2 in the supplemental material, and inserted into plasmid pET24b (Novagen). The resultant pET24b-derived plasmids were transformed into *E. coli* BL21-Gold(DE3), and the transformants were grown at 24°C at 200 rpm for 24 h (to produce the His-tagged RipAY and YER163c proteins) and at 30°C at 180 rpm for 24 h (to produce thioredoxins from various organisms) in Overnight Express Instant LB medium (Novagen) containing 20 µg/ml kanamycin. Cells were collected by centrifugation, washed twice with distilled water (DW), and resuspended in 0.1 volume of 100 mM Tris-HCl (pH 8.0) buffer containing 0.5 M NaCl for His-tagged proteins and 50 mM Tris-HCl (pH 8.0) buffer for other proteins without the His tag. The cell suspension was sonicated for successful cell lysis, and then the resulting lysate was centrifuged to remove cell debris. The resulting supernatant was collected and used as crude lysate for *in vitro* assay. His-tagged proteins were further purified from the supernatant using Talon metal affinity resin (Clontech, Palo Alto, CA) and PD-10 desalting columns (GE Healthcare, Marlborough, MA).

### Measurement of GSH level in yeast strains.

Yeast strains harboring pYC2/CT expressing the RipAY, RipAY E216Q, and YER163 proteins were incubated overnight at 30°C in SC −Ura medium supplemented with 2% glucose (repressing medium). Cells were collected by centrifugation, washed twice with DW, reinoculated in fresh SC −Ura medium supplemented with 2% galactose and 1% raffinose (expression medium) at an optical density at 600 nm (OD_600_) of 0.5, and further incubated at 30°C. Cells were collected every 2 h by centrifugation, washed twice with phosphate-buffered saline (PBS), and diluted with Y-PER reagent (Pierce, Rockford, IL) in appropriate volumes. Then a clear lysate (soluble protein fraction) was prepared in accordance with the manufacturer’s instructions, and the GSH level was measured with the GSH-Glo glutathione assay kit (Promega, Madison, WI). The total protein concentration of the lysate was also measured with a protein assay kit (Bio-Rad, Hercules, CA). The average GSH level (nanomoles of GSH per microgram of total soluble protein) was calculated and compared with that from yeast cells harboring the pYC2/CT empty vector.

### GSH degradation assay.

GSH (10 mM) was incubated with 3 µg of the RipAY or YER163c protein for 60 min at 30°C in 100 µl of reaction mixture containing 50 mM Tris-HCl (pH 8.0). The reaction mixture was then incubated at 95°C for 5 min to inactivate the enzyme. Yeast and plant extracts and *E. coli* lysates were added to the reaction mixture when necessary for each assay. The GSH level of the reaction mixture was measured with the GSH-Glo glutathione assay kit (Promega). Yeast extracts were prepared as described below. Plant extracts were prepared as follows. Briefly, leaf disks taken from *Arabidopsis* Col-0 plants were frozen in an Eppendorf tube in liquid nitrogen and then ground by shaking with tungsten beads using an SH-48 sample smasher (Kurabou, Osaka, Japan). Then 50 mM Tris-HCl (pH 8.0) buffer was added to the samples, and the mixture was thoroughly vortexed. Cell debris was removed by centrifugation, and the resulting supernatant was collected in new tubes and used for the assay as a plant extract. The purified recombinant proteins of yeast TRX2 thioredoxin (Oriental Yeast, Tokyo, Japan) and *E. coli* thioredoxin (Sigma, St. Louis, MO) were added to the reaction mixture at appropriate concentrations.

### Identification of degradation products by LC-MS.

To identify degradation products of GSH by using LC-MS, the products were confirmed by the selected-ion-monitoring (SIM) mode. The *m*/*z* values of [M+H]^+^ were adopted for the LC-MS-SIM. The LC-MS system consisted of a high-performance liquid chromatography (HPLC) device, LC-30AD (Shimadzu, Kyoto, Japan), and an MS detector with an electrospray ion source and quadrupole analyzer, LCMS-2020 (Shimadzu). LC-MS was carried out using an InertSustain column (4.6 by 50 mm; GL Sciences, Tokyo, Japan). The elution was performed with 0.1% formic acid for 5 min; the flow rate, column temperature, and injection volume were 0.5 ml/min, 35°C, and 5 µl, respectively. All samples were injected by 5× dilution with 0.1% formic acid. GSH (1 mM) (Sigma), 5-oxoproline (Sigma), and Cys-Gly (Sigma) were used as authentic standards.

### Preparation of yeast lysate and purification of yeast protein that activates RipAY.

*S. cerevisiae* YPH499 (62) was incubated for 24 h at 30°C in SC glucose medium supplemented with 2% glucose. Cells were collected by centrifugation, washed twice with 25 mM Tris-HCl (pH 8.0), and mechanically lysed using glass beads. The resulting lysate was centrifuged to remove cell debris, and the obtained supernatant was collected in new tubes and heat treated for 20 min at 95°C. Then heat-denatured proteins were removed by centrifugation, and the resulting supernatant was collected in new tubes and used as a yeast extract. For the purification of yeast proteins, the collected supernatant was dialyzed using a Vivaspin 100-kDa-cutoff spin column (GE Healthcare), and the flowthrough was concentrated using a Vivaspin 10-kDa-cutoff spin column (GE Healthcare) to collect 10- to 100-kDa yeast proteins. The proteins were dissolved in 20 mM Tris-HCl (pH 8.0) containing 150 mM NaCl and fractionated by preparative gel filtration chromatography using a Superdex 200 10/300-Gl column (GE Healthcare) with the same buffer at a flow rate of 0.5 ml/min with an Äkta fast protein liquid chromatography (FPLC) apparatus (GE Healthcare). Each 1-ml fraction was collected, dialyzed using an Amicon Ultra 10-kDa-cutoff spin column (Millipore, Billerica, MA), and assayed for its capability to activate RipAY. The active protein fractions were mixed and dissolved in 25 mM Tris-HCl (pH 8.0), loaded onto the Mono Q 5/50-Gl column (GE Healthcare) that had been preequilibrated with 25 mM Tris-HCl (pH 8.0) dialysis buffer, and eluted using a linear gradient of NaCl from 0 to 0.3 M at a flow rate of 1.0 ml/min with the Äkta FPLC apparatus. Each 1-ml fraction was collected with the Äkta FPLC apparatus, dialyzed using the Amicon Ultra 10-kDa-cutoff spin column, and assayed for its capability to activate RipAY. Each dialyzed fraction was loaded onto an SDS-PAGE gel and stained with colloidal Coomassie blue.

### Identification of yeast proteins by LC-MS/MS.

Protein bands of interest were excised and digested in-gel with sequencing-grade, modified trypsin (Promega). The resulting tryptic peptides were separated and analyzed by nano-liquid chromatography-electrospray ionization-quadrupole time of flight tandem mass spectrometry (NanoLC-ESI-Q-TOF MS/MS), and the collected data were analyzed by database search using the MASCOT program. Only significant hits as defined by MASCOT probability analysis were considered initially.

### *Agrobacterium*-mediated transient expression in *N. benthamiana* leaves.

The *ripAY* and *ripAY* E216Q gene cassettes were PCR amplified with HA coding sequences using the primer sets P3211 and P3236 and were inserted into the binary vector pEl2Ω-MCS (63) using an In-Fusion cloning kit (Clontech). The resulting plasmids were transformed into *A. tumefaciens* strain GV3101, and leaves from 4- to 5-week-old *N. benthamiana* plants were infiltrated with bacteria (OD_600_ of 0.5) suspended in infiltration buffer (10 mM MgCl_2_, 10 mM MES [morpholineethanesulfonic acid], 150 µg/ml acetosyringone). The inoculated plants were kept at 25°C in a plant incubator.

### Measurement of GSH level of *N. benthamiana* leaves.

Leaf disks (20 mg) were taken 48 h after agroinfiltration and were frozen in 2-ml Eppendorf tubes in liquid nitrogen. The frozen samples were ground into powder using an SH-48 sample smasher (Kurabou, Osaka, Japan). The powder was homogenized in 100 µl of 5% trichloroacetic acid (TCA), and the homogenate was centrifuged at 20,000 × *g* for 20 min at 4°C. The supernatant was collected in a new tube, extracted three times with diethyl ether to remove TCA, and assayed with a GSH-Glo glutathione assay kit to measure the GSH level.

### GFP microscopy.

*ripAY* and *ripAY* E216Q were fused in frame into *gfp* of pEl2Ω-gfp to construct a binary vector expressing the GFP-RipAY and GFP-RipAY E216Q proteins. The primer sets used for plasmid construction are listed in Table S2 in the supplemental material. The resulting plasmids were transformed into *A. tumefaciens* GV3101, and the transformants were used for experiments on transient expression in *N. benthamiana* leaves. Two days after infiltration, protoplasts were prepared from infiltrated leaves as described by D’Angelo et al. (64). The fluorescence of GFP and the autofluorescence of chloroplasts were observed by an FV1200 confocal laser scanning microscopy (Olympus, Tokyo, Japan).

### Measurement of ROS production level in *N. benthamiana* leaves.

The ROS production level was measured as described by Segonzac et al. (42) using L-012-mediated chemiluminescence instead of luminol and horseradish peroxidase. The ROS production level induced by 100 nM flg22 was measured by photon counting using an SH8000 microplate reader (Corona, Ibaraki, Japan)

### qRT-PCR.

Quantitative real-time PCR (qRT-PCR) was carried out as described by Nakano et al. (65). *NbEF1*α was used as an internal control for the normalization of transcript levels of *NbACRE132*, *NbPti5*, and *NbWRKY22*. The primer sets used for the qRT-PCR analysis are listed in Table S2 in the supplemental material.

### Construction of *R. solanacearum* Δ*gshAB* mutants.

For the construction of Δ*gshAB* mutants, a 2.7-kb BamHI fragment including the *amtB-gshA-gshB* region was PCR amplified from the genomic DNA of RS1000 using the primer sets P3065 and P3067 with BamHI sites and was inserted into pK18*mobsacB* (66). The resulting plasmid was doubly digested with SacII and NruI to delete the 0.9-kb fragment, including parts of *gshA* and *gshB*, and the 2.2-kb SacII-EcoRV streptomycin (Sm^r^) and spectinomycin (Spc^r^) resistance gene cassette was inserted in it to yield pK18*mobsacB* carrying the Δ*gshAB*::Sm^r^/Spc^r^ construct. The resultant plasmid was used to introduce the Δ *gshAB*::Sm^r^/Spc^r^ mutation into *R. solanacearum* strains by the marker-exchange method using *E. coli* S17-1 (66).

### Disk diffusion sensitivity tests and growth curve analysis.

*R. solanacearum* strains were incubated overnight in BG medium at 28°C. Cells were collected by centrifugation, washed twice with DW, and further incubated in 1/4 M63 glucose minimal medium at 28°C for 5 h. Cells were recollected by centrifugation, washed twice with DW, and resuspended in DW to yield an OD_600_ of 0.5 (~5 × 10^8^ CFU/ml). Cell suspensions were spread on BG plates, a piece of filter paper with a diameter of 1 cm was placed at the center of the plate, and then 10 µl of 3% H_2_O_2_ or 100 mM methylglyoxal was instilled into the piece of filter paper. After a 1-day incubation at 28°C, the radius of the inhibition zone of bacterial growth that was formed around the filter paper was measured. For growth analysis, cells incubated in 1/4 M63 glucose minimal medium at 28°C for 5 h were resuspended in the same medium at an OD_600_ of 0.1 to prepare an initial culture.

## SUPPLEMENTAL MATERIAL

Text S1  Supplemental references. Download Text S1, PDF file, 0.1 MB

Figure S1  The expressions of RipA4, RipA5, RipI, and RipAN did not affect the GSH level in yeast cells. The GSH level in yeast cells was measured 12 h after induction. The relative GSH levels (percentages) are shown in comparison with that in the vector control. The data are means ± SD from three independent experiments. Download Figure S1, PDF file, 0.9 MB

Figure S2  The expression of N-terminal His-tagged RipAY inhibited yeast growth. Yeast strains harboring pYC2/CT and pYC2/CT expressing N-terminal His-tagged RipAY were streaked on the repressing (Glc) and inducing (Gal) media and incubated at 30°C. Photographs were taken after 2 and 3 days of incubation, respectively. Download Figure S2, PDF file, 2 MB

Figure S3  MASCOT search results. Peptides derived from a tryptic digestion of a protein band of interest were separated and analyzed by NanoLC-ESI-Q-TOF MS/MS. The collected data were analyzed by database search using the MASCOT program. Download Figure S3, PDF file, 0.4 MB

Figure S4  Growth of *R. solanacearum* strains in stem of eggplants. A set of four plants were inoculated with 5 × 10^3^ cells of each *R. solanacearum* strain, RS1002 (*ripAY*^+^) and RS1700 (Δ*ripAY*), using a stem-cutting method as described previously (19). The *R. solanacearum* Δ*ripAY* strain RS1700 was constructed by a standard gene disruption procedure using a marker exchange plasmid, pK18*mobsacB*. The primer sets used for construction of pK18*mobsacB* carrying the Δ*ripAY* construct are listed in Table S2 in the supplemental material. Download Figure S4, PDF file, 0.9 MB

Table S1  Bacterial and yeast strains. Table S1, PDF file, 0.1 MB

Table S2  Primer sets used in this study. Table S2, PDF file, 0.1 MB
