# Supplementary material for: Ralstonia solanacearum Type III Effector RipAY Is a Glutathione-Degrading Enzyme That Is Activated by Plant Cytosolic Thioredoxins and Suppresses Plant Immunity
Source: mBio. 2016 Apr 12;7(2):e00359-16. doi: 10.1128/mBio.00359-16 (PMC4959522; doi:10.1128/mBio.00359-16)
Supplement: Text S1 — Supplemental references. Download [file mbo002162778s1.pdf]

Supplemental References

S1. **Sambrook J, Fritsch EF, Maniatis T.** 1989. Molecular cloning: a laboratory manual, 2nd ed. Cold Spring Harbor Laboratory Press, Cold Spring Harbor, N.Y.

S2. **Simon R, Priefer U, Pühler A.** 1983. A broad host range mobilization system for *in vivo* genetic engineering: Transposon mutagenesis in Gram-negative bacteria. Bio/Technology **1:**784–791.

S3. **Lamblin F**, **Saladin G**, **Dehorter B**, **Cronier D**, **Grenier E**, **Lacoux J**, **Bruyant P**, **Lainé E**, **Chabbert B**, **Girault F**, **Monties B**, **Morvan C**, **David H, David A.** 2001. Overexpression of a heterologous *sam* gene encoding S-adenosylmethionine synthetase in flax (*Linum usitatissimum*) cells: consequences on methylation of lignin precursors and pectins. Physiol Plant **112:**223–232.
