# Supplementary material for: Ralstonia solanacearum Type III Effector RipAY Is a Glutathione-Degrading Enzyme That Is Activated by Plant Cytosolic Thioredoxins and Suppresses Plant Immunity
Source: mBio. 2016 Apr 12;7(2):e00359-16. doi: 10.1128/mBio.00359-16 (PMC4959522; doi:10.1128/mBio.00359-16)
Supplement: Figure S1 — The expressions of RipA4, RipA5, RipI, and RipAN did not affect the GSH level in yeast cells. The GSH level in yeast cells was measured 12 h after induction. The relative GSH levels (percentages) are shown in comparison with that in the vector control. The data are means ± SD from three independent experiments. Download [file mbo002162778sf1.pdf]

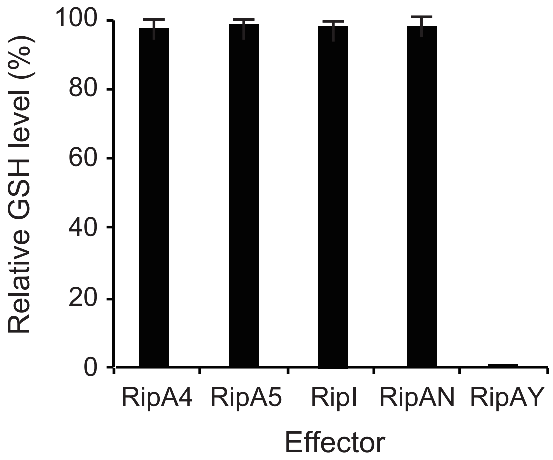

Fig. S1. The expressions of RipA4, RipA5, RipI and RipAN did not affect the GSH level in yeast cells. The GSH level in yeast cells is measured 12 hours after induction. The relative GSH levels (%) are shown in comparison with that in the vector control. The data are the mean ± SD of three independent experiments.
