# Supplementary material for: Ralstonia solanacearum Type III Effector RipAY Is a Glutathione-Degrading Enzyme That Is Activated by Plant Cytosolic Thioredoxins and Suppresses Plant Immunity
Source: mBio. 2016 Apr 12;7(2):e00359-16. doi: 10.1128/mBio.00359-16 (PMC4959522; doi:10.1128/mBio.00359-16)
Supplement: Figure S2 — The expression of N-terminal His-tagged RipAY inhibited yeast growth. Yeast strains harboring pYC2/CT and pYC2/CT expressing N-terminal His-tagged RipAY were streaked on the repressing (Glc) and inducing (Gal) media and incubated at 30°C. Photographs were taken after 2 and 3 days of incubation, respectively. Download [file mbo002162778sf2.pdf]

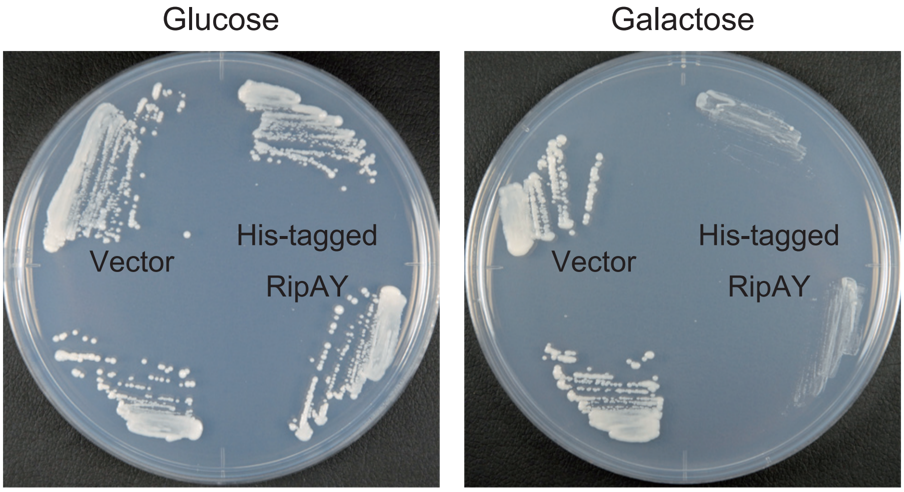

Fig. S2. The expression of N-terminal His-tagged RipAY inhibited yeast growth. Yeast strains harboring pYC2/CT and pYC2/CT expressing N-terminal His-tagged RipAY is streaked on the repressing (Glc) and inducing (Gal) media, and incubated at 30 ° C; photographs were taken after 2 and 3 days of incubation, respectively.
