# Supplementary material for: Ralstonia solanacearum Type III Effector RipAY Is a Glutathione-Degrading Enzyme That Is Activated by Plant Cytosolic Thioredoxins and Suppresses Plant Immunity
Source: mBio. 2016 Apr 12;7(2):e00359-16. doi: 10.1128/mBio.00359-16 (PMC4959522; doi:10.1128/mBio.00359-16)
Supplement: Figure S3 — MASCOT search results. Peptides derived from a tryptic digestion of a protein band of interest were separated and analyzed by NanoLC-ESI-Q-TOF MS/MS. The collected data were analyzed by database search using the MASCOT program. Download [file mbo002162778sf3.pdf]

MATRIX SCIENCE **Mascot Search Results**

User :
Email :
Search title :
MS data file :
Database : NCBInr 20141019 (51471198 sequences; 18460256539 residues)
Taxonomy : Saccharomyces Cerevisiae (baker's yeast) (50168 sequences)
Timestamp :
Protein hits : gi|6321879 Rtc3p [Saccharomyces cerevisiae S288c]
gi|122920148 Chain A, Crystal Structure Of Oxidized Form From Saccharomyces Cerevisiae
gi|2131127 RPS21A [Saccharomyces cerevisiae]
gi|6320275 Fmp16p [Saccharomyces cerevisiae S288c]
gi|323308207 Mrp17p [Saccharomyces cerevisiae FostersO]
gi|323349895 Ccr4p [Saccharomyces cerevisiae Lalvin QA23]
gi|6321935 Chs7p [Saccharomyces cerevisiae S288c]

Mascot Score Histogram

Ions score is -10*Log(P), where P is the probability that the observed match is a random event.
Individual ions scores > 28 indicate identity or extensive homology (p<0.05).
Protein scores are derived from ions scores as a non-probabilistic basis for ranking protein hits.

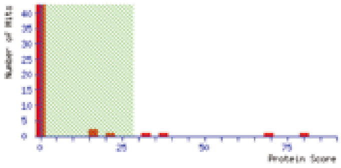

Peptide Summary Report

Format As: Peptide Summary — Help
Significance threshold p< 0.05 — Max. number of hits AUTO
Standard scoring — MudPIT scoring — Ions score or expect cut-off 0 — Show sub-sets 0
Show pop-ups — Suppress pop-ups — Sort unassigned Decreasing Score — Require bold red
Preferred taxonomy All entries

Select All — Select None — Search Selected — Error tolerant

1. gi|6321879 Mass: 12002 Score: 80 Matches: 2(2) Sequences: 2(2) emPAI: 0.62
Rtc3p [Saccharomyces cerevisiae S288c]
Check to include this hit in error tolerant search

Query	Observed	Mr(expt)	Mr(calc)	Delta	Miss	Score	Expect	Rank	Unique	Peptide
104	495.2290	988.4434	988.4825	-0.0391	0	67	9.8e-06	1	U	R.GAEGELGAASK.A
110	511.2291	1020.4437	1020.4876	-0.0439	0	32	0.028	1	U	K.AQVENEFGK.G

Proteins matching the same set of peptides:
gi|30749784 Mass: 14164 Score: 80 Matches: 2(2) Sequences: 2(2)
Chain A, Solution Nmr Structure Of Protein Yhr087w From Saccharomyces Cerevisiae. Northeast Structural Genomics Consortium Target Ytyst425

2. gi|122920148 Mass: 12264 Score: 67 Matches: 6(2) Sequences: 3(2) emPAI: 0.61
Chain A, Crystal Structure Of Oxidized Form From Saccharomyces Cerevisiae
Check to include this hit in error tolerant search

Query	Observed	Mr(expt)	Mr(calc)	Delta	Miss	Score	Expect	Rank	Unique	Peptide
85	351.6791	701.3437	701.3708	-0.0271	0	9	0.8	1	U	K.QAIASNV.-
97	466.7307	931.4468	931.4871	-0.0403	0	38	0.012	1	U	K.MIAPMIEK.F
99	470.2680	938.5214	938.5549	-0.0335	0	44	0.0028	1	U	R.VVGANPAAIK.Q
100	474.7274	947.4403	947.4820	-0.0416	0	(7)	14	1	U	K.MIAPMIEK.F + Oxidation (M)
101	474.7291	947.4436	947.4820	-0.0384	0	(24)	0.27	1	U	K.MIAPMIEK.F + Oxidation (M)
102	482.7255	963.4364	963.4769	-0.0405	0	(17)	1.1	1	U	K.MIAPMIEK.F + 2 Oxidation (M)

Proteins matching the same set of peptides:
gi|323714537 Mass: 11238 Score: 67 Matches: 6(2) Sequences: 3(2)
Chain A, Crystal Structure Of Mxr1 From Saccharomyces Cerevisiae In Complex With Trx2
gi|349578415 Mass: 11341 Score: 67 Matches: 6(2) Sequences: 3(2)
K7_Trx2p [Saccharomyces cerevisiae Kyokai no. 7]
gi|383875712 Mass: 12248 Score: 67 Matches: 6(2) Sequences: 3(2)
Chain B, Crystal Structure Of Peroxiredoxin Ahp1 From Saccharomyces Cerevisiae In Complex With Thioredoxin Trx2
gi|398366149 Mass: 11311 Score: 67 Matches: 6(2) Sequences: 3(2)
thioredoxin TRX2 [Saccharomyces cerevisiae S288c]

3. gi|2131127 Mass: 8882 Score: 38 Matches: 1(1) Sequences: 1(1) emPAI: 0.38
RPS21A [Saccharomyces cerevisiae]
Check to include this hit in error tolerant search

Query	Observed	Mr(expt)	Mr(calc)	Delta	Miss	Score	Expect	Rank	Unique	Peptide
103	486.2583	970.5020	970.5447	-0.0427	0	38	0.012	1	U	R.LAQNDGLLK.N

Proteins matching the same set of peptides:
gi|6322325 Mass: 9811 Score: 38 Matches: 1(1) Sequences: 1(1)
ribosomal 40S subunit protein S21B [Saccharomyces cerevisiae S288c]
gi|256272110 Mass: 9910 Score: 38 Matches: 1(1) Sequences: 1(1)
Rps21bp [Saccharomyces cerevisiae JAY291]
gi|398365233 Mass: 9797 Score: 38 Matches: 1(1) Sequences: 1(1)

Fig. S3. Mascot search results. Peptides derived from a tryptic digestion of a protein band of interest were separated and analyzed by nanoLC-ESI-Q-TOF mass spectrometry (MS/MS). The collected data were analyzed by database search using the MASCOT program.
