# Supplementary material for: Ralstonia solanacearum Type III Effector RipAY Is a Glutathione-Degrading Enzyme That Is Activated by Plant Cytosolic Thioredoxins and Suppresses Plant Immunity
Source: mBio. 2016 Apr 12;7(2):e00359-16. doi: 10.1128/mBio.00359-16 (PMC4959522; doi:10.1128/mBio.00359-16)
Supplement: Figure S4 — Growth of R. solanacearum strains in stem of eggplants. A set of four plants were inoculated with 5 × 10^3^ cells of each R. solanacearum strain, RS1002 (ripAY+) and RS1700 (ΔripAY), using a stem-cutting method as described previously (19). The R. solanacearum ΔripAY strain RS1700 was constructed by a standard gene disruption procedure using a marker exchange plasmid, pK18mobsacB. The primer sets used for construction of pK18mobsacB carrying the ΔripAY construct are listed in Table S2 in the supplemental material. Download [file mbo002162778sf4.pdf]

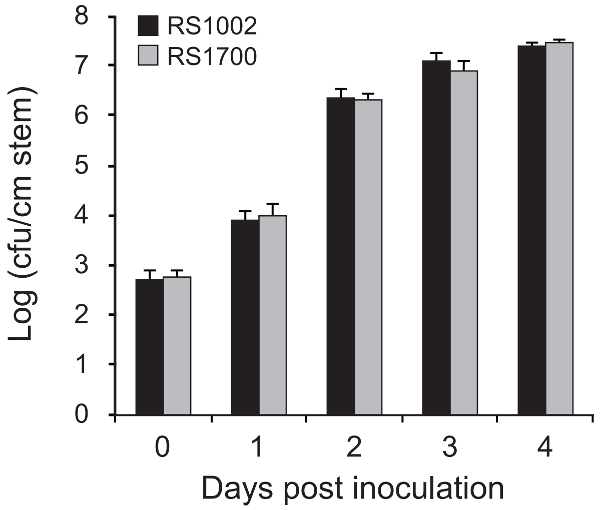

Fig. S4. Growth of *R. solanacearum* strains in stem of eggplants. A set of four plants were inoculated with 5 x 10^3 cells of each *R. solanacearum* strain, RS1002 (*ripAY*$^+$) and RS1700 (Δ*ripAY*), using a stem-cutting method as described previously (19). *R. solanacearum* Δ*ripAY* strain RS1700 is constructed by a standard gene disruption procedure using a marker exchange plasmid pK18*mobsacB*. Primer sets used for construction of pK18*mobsacB* carrying the Δ*ripAY* construct were listed in Table S2.
