# Supplementary material for: Ralstonia solanacearum Type III Effector RipAY Is a Glutathione-Degrading Enzyme That Is Activated by Plant Cytosolic Thioredoxins and Suppresses Plant Immunity
Source: mBio. 2016 Apr 12;7(2):e00359-16. doi: 10.1128/mBio.00359-16 (PMC4959522; doi:10.1128/mBio.00359-16)
Supplement: Table S1 — Bacterial and yeast strains. [file mbo002162778st1.pdf]

TABLE S1 Bacterial and yeast strains

Strain	Relevant genotype	Reference
Ralstonia solanacearum		
RS1002	RS1000 Nal^r (phylotype I, biovar 4)	(19)
RS1700	RS1002 Δ*ripAY*	This study
RS1701	RS1002 Δ*gshAB*::Sm^r/Spc^r	This study
Escherichia coli		
JM109	*E. coli* K12 *recA1 supE44 endA1 hsdR17 gyrA96 relA1* Δ(*lac-proAB*) F' [*traD36 proAB*$^+$ *lacI*^{q} *lacZ*ΔM15]	(S1)
BL21-Gold (DE3)	*E. coli* B F$^-$ *dcm*+ Hte *ompT hsdS*(r_B- m_B-) *gal* λ(DE3) *endA* Tet^r	Agilent Technologies
S17-1	*E. coli* K12 *thi pro hsdR*$^-$ *hsdM*$^+$ *recA* [chr::RP4-2-Tc::Mu-Km::Tn*7*]	(S2)
Agrobacterium tumefaciens		
GV3101	$Gm^{r,}$ Rif^r	(S3)
Saccharomyces cerevisiae		
YPH499	*MAT*a *ura3-52 lys2-801 ade2-101 trp1-63 his3-200 leu2-1*	(62)
KDY97.1a	*MAT*a *his*4 *HMLa leu2-3, 112 rme1 trp1 ura3-52* Δ*cpr1*::*LEU2* Δ*cpr2*::*TRP1 cpr3*::*HIS3* Δ*cpr4*::*URA3* Δ*cpr5*::*LEU2* Δ*cpr6*::*G418* Δ*cpr7*::*G418* Δ*cpr8*::*G418*	(59)
