# Supplementary material for: Ralstonia solanacearum Type III Effector RipAY Is a Glutathione-Degrading Enzyme That Is Activated by Plant Cytosolic Thioredoxins and Suppresses Plant Immunity
Source: mBio. 2016 Apr 12;7(2):e00359-16. doi: 10.1128/mBio.00359-16 (PMC4959522; doi:10.1128/mBio.00359-16)
Supplement: Table S2 — Primer sets used in this study. [file mbo002162778st2.pdf]

Table S2 Primer sets used in this study[a]

Target gene	Comment for product	Primer sets	Primer sequence (5'-3')
Construction of gene cassettes			
ripAY	Native form, for expression in pYC2/CT	P1002	5'-GCTCTAGAATGGAAAGAATCTCGACAAATG-3'
		P1003	5'-GGACTAGTTCAGTCGGGTTTGGGCTGCGTG-3'
ripAY	N-terminal His-tagged form, for expression in pET24b	P3063	5'-GCATATGGGCAGCAGCCATCACCATCATCACCACAGCCAGATGGAAAGAATCTCGACAAATGC-3'
		P3037	5'-GGACTAGTTCACTCGGTCGTCCGGATCAAG-3'
ripAY	N-terminal HA-tagged form, for expression in pEl2Ω-MCS, In-Fusion cloning	P3211	5'-ATTCGGTACCACTAGATGTACCCATACGATGTTCCA-3'
		P3236	5'-ATTCGAGCTCACTAGTCAGTCGGGTTTGGGCTG-3'
ripAY	GFP fusion, for expression in pEl2Ω-*gfp*, In-Fusion cloning	P3237	5'-ATGGACGAGCTGTACATGGAAAGAATCTCGACAAA-3'
		P3238	5'-CCGCTTTACTTGTACTCAGTCGGGTTTGGGCTGCG-3'
YER163c	Native form, for expression in pYC2/CT	P3042	5'-GACTAGTATGACTAATGACAACAGTGGTAT-3'
		P3043	5'-GACTAGTTCACCTGTATTTATTTACAGTC-3'
YER163c	N-terminal His-tagged form, for expression in pET24b	P3064	5'-GCATATGGGCAGCAGCCATCACCATCATCACCACAGCCAGATGACTAATGACAACAGTGGTAT-3'
		P3040	5'-GGCGGCCGCCCTGTATTTATTTACAGTCTC-3'
RTC3	Native form, for expression in pET24b	P3078	5'-GGCATATGTCTACTGTAACCAAATACT-3'
		P3079	5'-GGGAATTCTCAATTGTAGGCTTTGGTTCCG-3'
TRX2	Native form, for expression in pET24b	P3084	5'-GGCATATGGTCACTCAATTAAAATCCG-3'
		P3085	5'-GGGAATTCCTATACGTTGGAAGCAATAGCT-3'
RsTrxA	Native form, for expression in pET24b	P3098	5'-GGCATATGAGCGAACAGATCAAGTATG-3'
		P3099	5'-GGGAATTCTTAGAGGTGGCTGTCCAGGAAG-3'
AtTRX-h1	Native form, for expression in pET24b	P3102	5'-GGCATATGGCTTCGGAAGAAGGACAAG-3'
		P3103	5'-GGGAATTCTTAAGCCAAGTGTTTGGCAATG-3'
AtTRX-h2	Native form, for expression in pET24b	P3104	5'-GGCATATGGGAGGAGCTTTATCAACTG-3'
		P3105	5'-GGGAATTCTTATGCTCTGAGTTTGCTAACT-3'
AtTRX-h3	Native form, for expression in pET24b	P3106	5'-GGCATATGGCCGCAGAAGGAGAAGTTA-3'
		P3107	5'-GGGAATTCTCAAGCAGCAGCAACAACTGTC-3'
AtTRX-h4	Native form, for expression in pET24b	P3108	5'-GGCATATGGCGGCAGAAGAGGGTCAAG-3'
		P3109	5'-GGGAATTCTTACGCAGTTGTAACACCAGTA-3'
AtTRX-h5	Native form, for expression in pET24b	P3110	5'-GGCATATGGCCGGTGAAGGAGAAGTGA-3'
		P3111	5'-GGGCGGCCGCTCAAGCAGAAGCTACAAGACC-3'

AtTRX-h7	Native form, for expression	P3112	5'-GGCATATGGGTTCCAATGTTTCATCTG-3'
	in pET24b	P3113	5'-GGGAATTCTTAAACCCTATGTTGTTCAATC-3'
AtTRX-h8	Native form, for expression	P3114	5'-GGCATATGGGTGCTAACGTTTCTACTC-3'
	in pET24b	P3115	5'-GGGAATTCTCAGAAGAAGGATTGTGTGTAT-3'
AtTRX-h9	Native form, for expression	P3116	5'-GGCATATGGGTAGCTGCGTCTCTAAGG-3'
	in pET24b	P3117	5'-GGGAATTCTCAAGGCCGTTGAGGACTTTCA-3'
AtTRX-m1	Mature form, for expression	P3165	5'-GGCATATGGACACTGCTACAGGAATTCCAG-3'
	in pET24b	P3119	5'-GGGCGGCCGCTTACAAGAATTTGTTGATGCTG-3'
AtTRX-f1	Mature form, for expression	P3166	5'-GGCATATGGTTGGTCAGGTGACGGAGGTCG-3'
	in pET24b	P3121	5'-GGGAATTCTCATCCGGAAGCAGCAGACCTC-3'
AtTRX-x	Mature form, for expression	P3167	5'-GGCATATGGGCGGAATCAAAGAGATTGGAG-3'
	in pET24b	P3133	5'-GGGCGGCCGCTTAAGCAACAGATATTGAGT-3'
AtTRX-y1	Mature form, for expression	P3168	5'-GGCATATGGAAGCCAAGAAGCAGACATTTG-3'
	in pET24b	P3135	5'-GGGCGGCCGCCTATGGCTTCACTTTTAGAG-3'
AtTRX-z	Mature form, for expression	P3170	5'-GGCATATGGGCAAGTTTGTCAGAGAAGATT-3'
	in pET24b	P3171	5'-GGGAATTCTCACATCTCGTTGTCAATGATA-3'
AtTRX-o1	Mature form, for expression	P3169	5'-GGCATATGGAAAATGGTGTTGTTCTAGTGA-3'
	in pET24b	P3137	5'-GGGCGGCCGCTCACTTGTAGAGCTGTTCCA-3'
Site-directed mutagenesis			
ripAY	RipAY E216Q	P3044	5'-CGTGTCATCCGCCGGCAACTACTGGACGAG-3'
		P3045	5'-CTCGTCCAGTAGTTGCCGGCGGATGACACG-3'
ripAY	RipAYC328S	P3124	5'-CGCTTTGTCGAATCCAGCGAAACAGCCAAG-3'
		P3125	5'-CTTGGCTGTTTCGCTGGATTCGACAAAGCG-3'
AtTRX-h3	AtTRX-*h3* C39S/C42S	P3163	5'-TGCAACATGGAGCCCACCTAGCCGTTTCATTG-3'
		P3164	5'-CAATGAAACGGCTAGGTGGGCTCCATGTTGCA-3'
AtTRX-h5	AtTRX-*h5* C39S/C42S	P3149	5'-AGCATCATGGTCTCCACCTAGCCGTTTCATTG-3'
		P3150	5'-CAATGAAACGGCTAGGTGGAGACCATGATGCT-3'
Construction of R. solanacearum mutants			
gshAB	for introducing	P3065	5'-GGGGATCCTCGCCAACAAGGTGGCCGATG-3'
	Δ*gshAB*::Sm^r/Spc^r	P3067	5'-GGGGATCCCGCTCGGACAGCAGTTGCGCC-3
ripAY	for introducing Δ*ripAY*	P3032	5'-GGGAATTCCGGCCCGCCATGCGG-3'
upstream		P3033	5'-GGGGATCCGGCTGGCACCGTCGATCGGGA-3
ripAY	for introducing Δ*ripAY*	P3034	5'-GGGGATCCCGCCACTCGCCATTCTCTCGC-3'
downstream		P3035	5'-GGAAGCTTAGCCGATGCCGATAATGTCC-3
Quantitative real time PCR			

NbACRE132	NbACRE132-qRT-FW	5'- CAAACGGCGGCACAAGAC -3'
	NbACRE132-qRT-RV	5'- GATAACCGCCAGCGAAATTG -3'
NbEF1α	NbEF1α-qRT-FW	5'- CCTCAAGAAGGTTGGATACAAC -3'
	NbEF1α-qRT-RV	5'- TCTTGGGCTCATTAATCTGGTC -3'
NbPti5	NbPti5-qRT-FW	5'- CCTCCAAGTTTGAGCTCGGATAGT -3'
	NbPti5-qRT-RV	5'- CCAAGAAATTCTCCATGCACTCTGTC -3'
NbWRKY22	NbWrky22-qRT-FW	5'- CAAGGCATAAAGCAGACACAACA -3'
	NbWrky22-qRT-RV	5'- GCTTCTGACCACCGCATGT -3'

[a] The restriction sites used for cloning are underlined.
